# Comprehensive Approaches to Endometriosis Management and Targeted Strategies for Bowel Endometriosis

**DOI:** 10.3390/jcm15031040

**Published:** 2026-01-28

**Authors:** Arrigo Fruscalzo, Alexandre Vallée, Carolin Marti, François Pugin, Jean-Marc Ayoubi, Michael D. Mueller, Anis Feki

**Affiliations:** 1 Department of Gynecology and Obstetrics, University Hospital of Fribourg, 1708 Fribourg, Switzerland; carolin.marti@h-fr.ch (C.M.); jm.ayoubi@hopital-foch.com (J.-M.A.); anis.feki@h-fr.ch (A.F.); 2 Department of Gynecology and Obstetrics and Reproductive Medicine, Hôpital Foch, 75017 Paris, France; al.vallee@hopital-foch.com; 3 Department of General Surgery, University Hospital of Fribourg, 1708 Fribourg, Switzerland; francois.pugin@h-fr.ch; 4 Department of Gynecology and Obstetrics, University Hospital of Berne, 3010 Bern, Switzerland; michel.mueller@insel.ch

**Keywords:** bowel endometriosis, intestinal endometriosis, surgical morbidity, classification, nodule shaving, discoid excision, segmental intestinal resection, NOSE (natural orifice specimen extraction)

## Abstract

**Background**: Deep infiltrating endometriosis (DIE) and, in particular, bowel endometriosis stand out for their complexity. While surgery for bowel endometriosis has proven to be effective, there is a lack of standardization concerning the technique used and the reported outcomes. **Objectives**: The objective is to perform a review aiming to summarize the state of the art of bowel endometriosis and to point out the gaps to be addressed by future research. We also propose a novel classification of surgical procedures to fill these gaps and improve management. **Methods**: A literature search was performed on PubMed from inception to October 2025. **Results**: The following three major procedures for the excision of bowel endometriosis have been proposed: the nodule shaving, the discoid excision, and the segmental intestinal resection. One further technique, NOSE (natural orifice specimen extraction), can be applied for the removal of the specimen in cases of discoid or segmental resection. To reduce surgical morbidity, current data support the choice of most conservative surgical options, namely nodule dissection and discoid resection, as well as the use of nerve-sparing techniques in case of segmental resection. Nonetheless, there is little evidence concerning the indication and the most appropriate technique to be used, including their relative risks and benefits in terms of pain control, urinary and gastrointestinal function, risk of future relapse, and fertility outcomes. **Conclusions**: Significant barriers in comparing surgical outcomes due to unclear definitions, lack of standardization, and incomplete reporting are some of the most relevant issues frequently encountered. To fill these gaps, we propose a new classification system for bowel surgery that describes the dimension and the number of the lesions, as well as the type of surgical technique used, supplemented by the information if vaginal opening was necessary for complete lesion resection. This proposition aims to open a discussion on this topic and boost focused research to evaluate the utility of a new classification in clinical practice.

## 1. Introduction: Endometriosis, Still Many Questions to Be Answered

Endometriosis is a chronic, inflammatory, relatively common pathology affecting up to 10% of women in their reproductive age [1]. It is characterized by the presence of endometrial glands and stroma outside the uterus and is responsible for chronic cyclic pain and infertility. Despite its benign nature, it has the potential to evolve, in some cases, into a debilitating illness, responsible for a considerable physical and social burden [2]. Unfortunately, it remains unclear how endometriosis develops and how different clinical appearances originate, making it very challenging to screen for the most severe cases. Despite these limitations, the awareness surrounding this pathology is steadily growing, including information campaigns at a social level and supporting general and specific medical competencies [3]. Developing this alertness of endometriosis will help clinical and basic research toward a better understanding of this pathology.

Currently, the following three major forms of endometriosis are recognized: one giving rise to ovarian cysts, also called ovarian endometriosis; the superficial form, affecting the peritoneum, also called peritoneal endometriosis; and the form infiltrating the pelvic and other organs, also called deep infiltrating endometriosis [4]. In addition to these, there is a further form characterized by the presence of endometrial glands and stroma inside the uterine myometrium, also called adenomyosis [4].

Considering the different phenotypes of this pathology, it seems that these forms are probably defined by different underlying pathogenic mechanisms. Retrograde menstruation, coelomic metaplasia, and lymphatic or vascular dissemination are the most common mechanisms underpinning endometriosis [5]. However, beneath this evidence, it is reasonable to assume that specific endocrine, immunologic, and inflammatory processes are implicated in the development of the disease in some patients rather than others. This is the case for tissue transplantation by retrograde menstruation, which is a quite common phenomenon, more frequently observed than endometriosis is [6].

## 2. Materials and Methods

A literature search was performed on PubMed from inception to October 2025. The following keywords were used: “bowel endometriosis” OR “intestinal endometriosis”, “surgical morbidity”, “endometriosis AND classification”, “endometriosis AND nodule shaving” OR “discoid excision” OR “segmental intestinal resection”; “endometriosis AND NOSE (natural orifice specimen extraction)”, “adenomyosis AND classification”. The search was limited to the English language and to full-text articles. Reviews, case reports, and commentaries were excluded. Abstracts were screened by two authors (AFr and AV), and relevant articles were selected. In case of conflicts between reviewers, a third author (AFe) chaired a shared discussion and provided the final decision. The process of article selection is described according to the PRISMA flow chart [7].

## 3. Results

**Figure 1:**
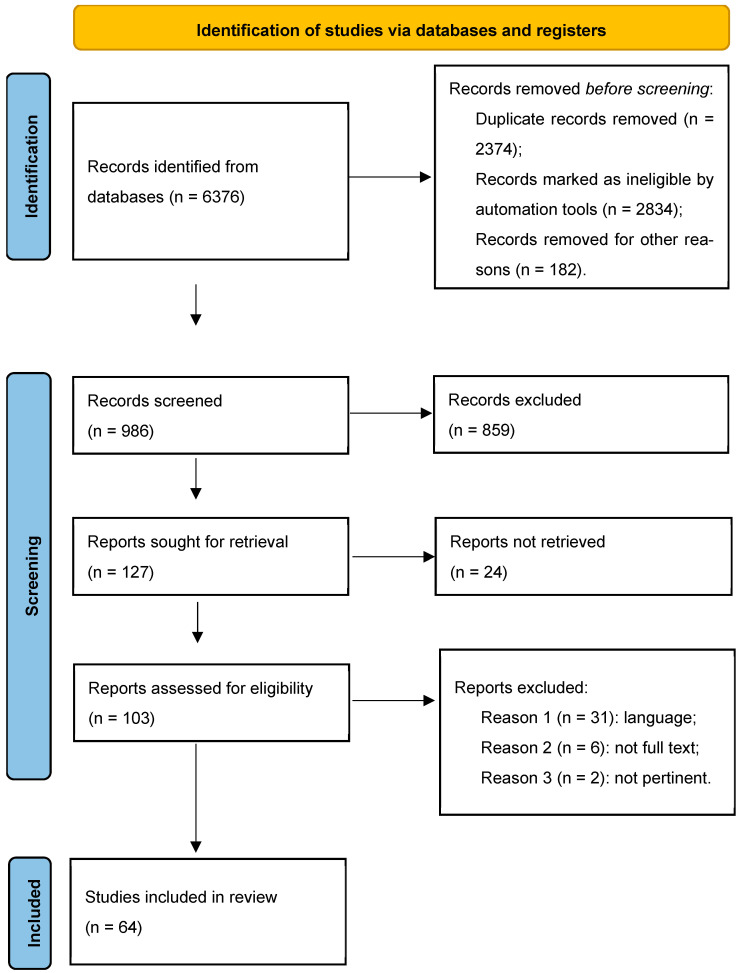
PRISMA flow diagram and article selection.

According to the chosen keywords, 6376 articles were first identified. After removing duplicate and ineligible articles, a total of 986 manuscripts were submitted for the first screening. In the further process excluding non-retrieved, non-pertinent reports, or reports not written in the English language, we finally identified 64 manuscripts included in the study (Figure 1).

### 3.1. Bowel Endometriosis: A Complex Clinical Entity

Beside the above-mentioned evidence, we would like to further stress current knowledge in endometriosis, particularly concerning deep endometriosis infiltrating the bowel. This form of endometriosis remains a hot topic in gynecologic research, as clinical and surgical management represent a major challenge for gynecologists and general surgeons. Many aspects remain unclear and research is still growing in this context.

Bowel endometriosis, indeed, stands out for its complexity and the severe complications it can cause if not properly managed [8]. While surgery for deep infiltrating endometriosis (DIE) has proven effective, it also carries a considerable risk of complications [9,10]. Numerous techniques for the excision of bowel endometriosis have been explored. However, the field lacks large, prospective randomized controlled trials (RCTs) [11]. Systematic reviews reveal significant barriers in comparing surgical outcomes due to unclear definitions, a lack of standardization, and incomplete reporting [12]. Thus, a concerted effort to standardize data collection in surgical trials for bowel endometriosis is urgently needed.

### 3.2. Epidemiology and Risk Factors

According to the current literature, endometriosis has been observed in 5 to 10% of women in reproductive age but can affect up to 50% of women experiencing infertility [13]. Deep endometriosis is generally accompanied by other forms of endometriosis, notably the peritoneal endometriosis and the presence of endometriomas, and less frequently, in about 6.5% of all cases, it occurs as an isolated form of endometriosis [14]. Finally, when looking into bowel endometriosis, it has been estimated that it accounts for 3% to 37% of all endometriosis cases [15]. Most of them concern the terminal part of the bowel, either the sigmoid or the rectum, accounting for more than 90% of all cases of intestinal endometriosis [16]. Risk factors for endometriosis include a genetic predisposition, early age at menarche, short menstrual cycles, lower BMI, nulliparity and congenital obstructive anomalies of the Müllerian system, and prolonged exposure to estrogen. Family history is one of the strongest predictors, with first-degree relatives having a significantly higher risk [17].

### 3.3. Pathogenesis

As already stated, pathogenesis of endometriosis remains elusive [18]. There is still a sharp debate surrounding the origins of DIE, including intestinal endometriosis, compared to the superficial one. Analogous to the original theory of Sampson of retrograde menstruation, it has been supposed that endometrial stem cells transported into the peritoneal cavity develop the potential to generate implants that can later in life further evolve into deep infiltrating endometriosis [19]. Another theory proposes that endometriotic clonal cells undergoing genetic or epigenetic modifications will develop peculiar molecular features characteristic of deep infiltrating endometriosis. Depending on the localization of these primary implants, the pathology will develop into different phenotypical features [19].

Unfortunately, a major limit of the current theories on the pathogenesis of DIE is that currently none of the proposed models solely explains the rise of the pathology. Indeed, it is true that typically rectosigmoid endometriosis originates in contact with retrocervical area or in the pouch of Douglas. Nonetheless, in many cases, there are isolated intestinal lesions that do not have any contact with other endometriosis lesions. Do these phenotypes have different pathogenetic mechanisms? The impression is that the pieces of the puzzle have not yet fallen into place. Bridging this gap of basic knowledge will help researchers better define future fields of clinical research.

Genetic factors play an important role in the development of endometriosis, particularly through polymorphisms in candidate genes involved in inflammation, estrogen metabolism, angiogenesis, and immune regulation [20]. In recent years, numerous genome-wide association studies and large-scale genetic association studies have identified susceptibility loci that contribute to disease risk, highlighting the complex, multifactorial nature of its pathogenesis [21]. Emerging genetic evidence suggests that deep infiltrating endometriosis, including rectal involvement, may represent a distinct and more heritable disease phenotype, with specific susceptibility loci showing stronger associations with advanced and invasive forms of endometriosis [22]. These findings highlight the importance of incorporating genetic evidence into the study of disease mechanisms, as they enhance our understanding of the biological pathways involved in endometriosis and help explain the variability in its clinical presentation.

Recent research has investigated the role of epigenetics in endometriosis. There is increasing evidence that DNA methylation plays a relevant role in developing specific phenotypes of endometriosis [23]. A recent study on epigenome-wide methylation profiling detected several genetic loci associated with the pathology [24]. Other studies focused on the role of miRNA in the emergence of endometriosis. miRNAs are gene expression regulators that act at the post-transcriptional level inducing mRNA degradation or inhibition of protein synthesis [25]. Detecting target genes responsible for endometriosis could shed new light on the understanding of the pathogenesis, thus helping to better address future research efforts.

### 3.4. Classification

Providing a feasible classification of endometriosis, including DIE, is one of the keys to future research in this field. It allows us to standardize clinical data, above all for comparing results of different management strategies.

Currently, the most accepted classification of deep endometriosis is the #Enzian classification [26]. For categorization purposes, it assumes that endometriosis can develop along the following three axes: the cranio–caudal axis (axis A), including the involvement of the rectovaginal space; the latero–lateral axis (axis B), including the uterosacral ligaments and the parametrium; and the antero–posterior axis (axis C), including the involvement of the rectosigmoid wall. This latter involvement was further classified in three categories according to the largest dimension of the endometriosis nodule, as C1 (<1 cm), C2 (between 1 and 3 cm), and C3 (>3 cm).

As one can see, this classification assumes that the lesion starts from the retrocervical area, infiltrating progressively the rectosigma. As already stated, this is, however, not always the case, given that isolated bowel lesions can also be found. In addition, this classification is related to macroscopic anatomical observations, describing the localization and dimensions of the nodules. However, a very challenging aspect of DIE is that macroscopic diagnosis can be very difficult, particularly if a pre-operative diagnostic imaging has been performed. Furthermore, it is well-known that one of the major constraints of this classification is the limited association between #Enzian classification and clinical presentation. This makes comparisons between different therapeutic approaches very challenging. Thus, identifying further clinically relevant features of the lesions, maybe including anatomopathological and molecular signature features in the future, will potentially help in better understanding the pathology and in refining stratification.

### 3.5. Clinical Presentation

It is generally recognized that the presence of deep infiltrating endometriosis greatly impacts clinical appearance of endometriosis, depending on the localization of the implants [27]. Intestinal endometriosis is typically associated with abdominal pain and various intestinal symptoms, such as dyschezia, bloating, constipation, or diarrhea, which may worsen during the menstrual cycle. Other symptoms can include cyclical rectal bleeding, urgency to defecate, and a sensation of incomplete evacuation. In rare cases, large endometriotic nodules in the large intestine can lead to significant narrowing of the intestinal lumen, causing occlusive or sub-occlusive symptoms [28].

Furthermore, there is evidence linking endometriosis to substantial fertility issues globally [29]. In longitudinal register data, natural fertility appears measurably diminished before diagnosis: in a nationwide Finnish cohort, the incidence rate ratio for a first live birth was 0.51 (95% CI 0.49–0.52) in women with endometriosis versus matched references, indicating roughly a halving of the rate of first births prior to surgical verification [30]. Population cohorts show a ~49% reduction in the rate of first live birth (IRR 0.51) prior to surgical diagnosis [30]. In ART, contemporary meta-analysis indicates ~15% lower implantation odds yet no clear LBR deficit, suggesting embryo competence may be preserved while uterine receptivity is subtly impaired. Assisted reproduction shows a nuanced pattern. A contemporary meta-analysis (2024) found that, while implantation is significantly reduced in women with endometriosis, live birth and clinical pregnancy rates do not differ materially from controls—consistent with a dominant uterine-receptivity signal rather than an embryo-intrinsic deficit. Specifically, implantation was lower despite no statistically significant differences in live birth, clinical pregnancy, or fertilization [31].

Despite this evidence, there is a large variability in clinical presentation, both related to the extent of endometriosis and its localization. It remains unclear how more detailed localization and extension of the lesions correlate to the symptoms and fertility. The attempt to describe the lesions by means of a classification that closely reflects the clinical presentation of pathology seems to still be suboptimal [32].

### 3.6. Non-Surgical Management Options for Bowel Endometriosis

Non-surgical treatment of bowel endometriosis aims to control pain and symptoms, primarily through hormonal therapies that suppress menstruation, alongside analgesics and lifestyle modifications. However, this approach is often effective for symptom relief only in less severe cases. Common options include combined oral contraceptives, progestogens (oral preparations or intrauterine systems), GnRH agonists, and antagonists [33]. Conservative management could be effective unless symptoms show signs of bowel obstruction, requiring surgical intervention for this reason. It is important to note that conservative treatments are generally not curative. Symptoms often return upon discontinuation. Furthermore, tight monitoring of the symptoms and of the characteristics of lesions is advised, as progression of the dimension of lesions can occur.

### 3.7. Surgical Management of Bowel Endometriosis

The surgical management of bowel endometriosis requires a nuanced approach that balances the need for radical intervention to prevent disease progression and recurrence while conserving bowel function and minimizing complications. It has been largely proven that surgical removal of the DIE lesions drastically improves the associated symptoms, including first dysmenorrhea, but also the dyspareunia, dyschezia, dysuria, and the chronic pelvic pain, as well as the fertility outcomes [34].

Concerning pain control, a recent prospective multicenter cohort study showed that both discoid full resection and segmental resection significantly improved gastrointestinal function-related quality of life in operated patients, also improving chronic pain and, in general, endometriosis-related quality of life profile [35]. Similar results were shown by Namazov et al. [36].

Concerning fertility, a systematic review and meta-analysis conducted in 2017 by Iversen et al. showed with some disappointment that, at that time, no randomized trial targeted at this issue had been performed [37]. The review was able to include only four retrospective and three prospective observational studies, indicating a possible positive effect on spontaneous pregnancy rates. Furthermore, a positive effect on IVF outcome could not be excluded. A 2025 systematic review/meta-analysis concluded that segmental colorectal resection is associated with a lower pregnancy rate compared with more conservative procedures (e.g., nodule shaving or discoid excision), supporting conservative, nerve-sparing strategies when future conception is a priority [38].

Despite these data, there is less evidence concerning the indication and the most appropriate technique to be used, including their relative risks and benefices. To date, the following three major techniques have been proposed: nodule shaving, discoid excision, and segmental resection. One further procedure, NOSE (natural orifice specimen extraction), can be applied for the removal of the specimen in case of segmental resection [39]. A sharp debate is still open concerning the choice of which option should be preferred in case of intestinal endometriosis. Each of these techniques has specific advantages and constraints. Thus, management should be adapted to the type of phenotype to optimize the clinical outcome for the patient.

### 3.8. Segmental Resection Versus Conservative Surgical Techniques

Segmental resection represented for a long time the gold-standard procedure for bowel endometriosis. It has been largely explored and described in colorectal surgery for benign and malign pathologies [40]. Recently, increasing attention has been given to conservative surgical techniques allowing sparing not only the intestinal wall, but also the vascular and nervous system belonging to the bowel [41]. It includes both the nodule shaving and the discoid excision. They were proposed to reduce short-term and long-term surgical complications [42]. Nonetheless, the results concerning radicality and the risk of endometriosis relapse are less clear [43].

#### 3.8.1. Nodule Shaving

Nodule shaving implies the shaving of the nodule to preserve rectal continuity, without opening the rectal wall. The nodule shaving can be performed when the bowel nodule interacts with other organs or structures, which typically occurs when the bowel is fixed to the uterine cervix, the adnexa, or the pelvic wall. This step can be necessary to further access the pelvis or, for example, in case a complete hysterectomy was planned. Compared to segmental resection, this technique allows also to preserve the pelvic autonomic nerves, which are key factors in the preservation of neurogenic control of rectal, bladder, and sexual function [44]. In experienced hands, it appears to be feasible in most cases, by using different devices like laser, cold scissors, ultrasound scalpel, or monopolar energy [45]. Also, according to several studies, pre-operative and post-operative complications appear to be reduced compared to segmental and discoid resection.

The most important limitation of nodule shaving is that in this case there is an elevated risk that nodules will be left in the bowel wall, sometimes only partially, sometimes almost completely. Thus, an accurate pre-operative estimation of the bowel wall infiltration in these cases is mandatory; leaving some endometriosis tissue in the bowel wall could expose the patient to a relapse or progression of the pathology [46]. Furthermore, even though suture leakage, a fearsome complication of segmental and discoid resection, will be avoided in case of nodule shaving, a risk of rectal fistula of about 1.6% has been described [47].

#### 3.8.2. Discoid Excision

The discoid excision is considered a conservative technique, as it allows the conservation of most of the rectal wall, including its nerves and vessels. It consists of a full-thickness circular rectal wall excision, followed by a transversal suture of the resected margins with a stapler or manually. The nodule should be preliminarily shaved in case it infiltrates the vaginal fornix, the uterine torus, or the uterosacral ligaments [48]. This procedure seems to show favorable short- and long-term outcomes, being related to fewer post-operative complications in comparison to segmental resection, even though its complication rates remain slightly higher compared to those of nodule shaving.

The most important limitation of the discoid resection concerns the size of the nodule, which can limit the use of the stapler for the nodule resection and the automated suture of the residual intestinal wall. Some authors suggest not performing it for nodules larger than 3–4 cm [49]. Nonetheless, for larger nodules, a second resection with a stapler could be performed in some selected cases [50].

#### 3.8.3. Segmental Resection

Segmental resection consists of the full-thickness resection of the bowel segment affected by the nodule. Typically, severe complications following this technique are the stenosis of the anastomosis or the appearance of a low anterior rectal resection syndrome (LARS), linked to altered bowel function and reduced quality of life [51]. Thus, it appears that bowel resection could be reserved for cases of very large nodules with bowel stenosis, as well as multiple and/or posterior rectal lesions, where conservative methods might not be feasible.

The localization of the nodule is an important issue for segmental resection. Most nodules will concern the mid to low rectum, with the nodule generally located between 4 and 12 cm from the anal verge, even though a higher location is also possible. This anatomical landmark is of key importance, as leakage complications are encountered more frequently in lower anastomosis [52]. In addition, the size of the resection should be adapted to the size of the nodule and should be considered as a main predictor for post-operative gastrointestinal dysfunction. In case of multiple nodules, a multiple segmental resection could be technically feasible if at least 5 cm of healthy bowel separates the nodules. Nonetheless, complications are expected to increase proportionally in this case [53].

#### 3.8.4. NOSE Technique

This technique has been recently developed to avoid surgical specimen removal in case of discoid or segmental bowel resection via small laparotomy. This technique has the potential to reduce the supplementary morbidity related to the abdominal wall opening via laparotomy [54,55]. A recent randomized controlled trial showed similar outcomes in terms of bowel function, endometriosis-related as well as gastrointestinal-related quality of life and pain score compared to the classic technique [56]. However, results concerning advantages obtained with this technique are still scarce and need to be confirmed by future studies.

### 3.9. Clinical Outcomes of the Different Surgical Techniques

Several factors could influence the selection of the surgical technique used in the surgery of intestinal endometriosis. In addition to personal confidence with the technique and feasibility criteria described above, post-operative short- and long-term outcomes should guide surgeon’s choice and patient’s counseling.

#### 3.9.1. Perioperative Complications

As already stressed, it seems that nodule shaving has a lower rate of surgical complications. Abo et al. described that rectal shaving is associated with a lower risk of perioperative complications, according to the Clavien-Dindo classification, compared to disk excision and colorectal resection [57]. A recent systematic review and meta-analysis showed that conservative surgery was associated with similar rates of suture leakage, abscess formation, and rectovaginal fistulas. However, in the subgroup analysis, nodule shaving showed the shortest operative time, a lower rate of need for a stoma, and lower rate of rectal stenosis. Superposable results were found in another similar meta-analysis [46].

#### 3.9.2. Gastrointestinal Function

Long-term intestinal function is one of the most relevant issues in bowel endometriosis surgery. Whenever possible, a full-thickness segmental resection should be avoided in order the reduce the risk of post-operative function impairment, including the low anterior resection syndrome. Low anterior resection syndrome (LARS) encompasses a collection of several symptoms, such as fecal incontinence and urgency, that can impact the quality of life of a patient after rectal surgery. Specific scores have been developed in order to assess intestinal function after surgery, in particular the LARS score, which investigates the presence of fecal incontinence, urgency, and frequency [58]. Also, it is suggested to document the women’s quality of life, by using, for example, the Gastrointestinal Quality of Life Index (GIQLI), which has been developed for the assessment of quality of life (QOL) in diseases of the upper and lower GI tract [59]. It has been underlined that nerve-sparing techniques should always be provided to preserve intestinal function as much as possible [56]. Indeed, a similar incidence of gastrointestinal function impairment was found among women operated by laparoscopic disk excision and nerve- and vessel-sparing segmental resection. Results were confirmed in a more recent prospective multicenter study comparing full-thickness discoid resection and nerve- and vessel-sparing segmental bowel resection, where authors also showed that both techniques provided an improvement of gastrointestinal function. No conclusion can be drawn concerning nodule shaving as the data are still lacking [34].

#### 3.9.3. Voiding Dysfunction

Post-operative voiding dysfunction is one of the most common complications of colorectal surgery that can occur in up to 10% of all operated patients [60]. Vesale et al. in their meta-analysis assessed the incidence of post-operative voiding dysfunction in case of surgery, defined as the intermittent need for post-operative self-catheterization or urinary retention. Results showed that voiding disfunction was significantly less frequent in rectal shaving compared to both segmental and discoid resection, accounting for 2.4% in case of nodule shaving, compared to, respectively, 7.5% and 10.1% in case of discoid excision and segmental resection [60]. A more recent meta-analysis assessed the risk of permanent voiding disfunction, defined as the need for self-catheterization for at least more than 1 month. They found that both nodule shaving and discoid excision were associated with significantly less persistent voiding disfunction than segmental resection [61].

#### 3.9.4. Sexual Function

Sexual function is another important domain to consider in the context of bowel endometriosis surgery. Bowel endometriosis involving the rectovaginal space and the pouch of Douglas may cause severe pain during sexual intercourse, particularly in specific positions. Also, chronic pain and anticipation can lead to a reduced sexual desire and satisfaction. Several scores have been proposed in order to evaluate this impact in women’s sexual life. Sabbatsberg Sexual Self-Rating Scale is a 12-item self-assessment questionnaire that evaluates 6 subdomains, including sexual interest, sexual activity, satisfaction of sexual life, experience of sexual pleasure, orgasm capacity, and sexual relevancy [62]. The FSFI (Female Sexual Function Index) assesses sexual function across the following six domains: desire, arousal, lubrication, orgasm, satisfaction, and pain [63].

Evaluating sexual complaints due to endometriosis, specifically in case of intestinal endometriosis surgery, is of paramount importance in the choice of therapy to be proposed [64]. While hormonal treatments do not result in improvement of sexual symptoms, surgical excision of nodules (especially nerve-sparing techniques) generally improves sexual function [65]. The impact of intestinal function related to intestinal nodules and in case of nodule resection should also be assessed and considered during the shared decision-making process [66].

#### 3.9.5. Recurrence

Bowel endometriosis recurrence has been reported to affect up to 15.3% of treated patients by surgery [46]. However, it must be stressed that the definition of recurrence largely varies, ranging from a clinical and imaging-based diagnosis to surgical confirmation. Furthermore, this outcome is frequently not reported at all among clinical studies. A more recent meta-analysis highlighted, as expected, that there were fewer recurrences in the segmental resection and the discoid resection groups compared to the nodule shaving group [43].

#### 3.9.6. Fertility

Deep infiltrating endometriosis (DIE), including colorectal involvement, concentrates reproductive risk. For rectal DIE, pooled data support conservative, nerve-sparing procedures when fertility is prioritized as follows: pregnancy rates are ~36–49% lower after segmental resection vs. nodule shaving, while differences vs. discoid are uncertain; these choices must be balanced against recurrence and morbidity profiles [37]. Even though data suggest a less favorable fertility outcome for segmental resection, it is not clear if it is linked to the severity of the disease as a confounding factor or if it is due to the surgical trauma itself. Further research is needed to better elucidate these results. In case of concomitant ovarian endometriomas, caution should be paid to avoid detrimental effects on post-operative ovarian reserve. The use of bipolar energy for dissection and hemostasis should be avoided as much as possible and suturing should be performed over the application of a hemostatic sealant for hemostasis [67,68].

From an obstetrical point of view, women with endometriosis, especially severe DIE, face higher risks of preterm birth (~31% relative increase) and placenta previa (~63% relative increase), warranting anticipatory perinatal care [30,69]. Women treated for bowel endometriosis show higher rates of adverse pregnancy and neonatal outcomes than healthy controls, aligning with the broader literature linking endometriosis to obstetric complications [70]. In pooled analyses across conception modes, placenta previa risk is markedly elevated in overall endometriosis (34 studies, OR 2.84; 95% CI 2.47–3.26), with stronger associations in severe phenotypes such as rASRM stage III–IV and deep endometriosis [71].

Beside this evidence, a very recent systematic review and meta-analysis was performed by Vallée et al. [38]. The data showed a lower pregnancy rate in case of rectal resection for endometriosis compared with other types of surgery, such as nodule shaving. Thus, in patients seeking pregnancy, segmental resection could not be the best option, as it is characterized by a lower pregnancy rate compared with other types of surgery, such as nodule shaving [38]. Nonetheless, caution should be taken when interpreting these results, as worsened fertility outcomes could be biased by the severity of the DIE, which is generally more serious in case of segmental resection than in case of conservative surgery. Taken together, these data argue for earlier recognition to shorten time to pregnancy, individualized ART pathways that mitigate receptivity deficits, and shared decision-making on colorectal DIE surgery that weighs symptom control against quantifiable reproductive trade-offs [72].

### 3.10. Cost-Effectiveness, Burden on the Health System, and Access of Different Techniques

Endometriosis-related economic burden is a major issue to discuss when reviewing the surgical approach to bowel endometriosis. Direct health care costs and indirect productivity loss costs have been estimated to be similar to several chronic diseases like diabetes mellitus, Crohn’s disease, ankylosing spondylitis, or rheumatoid arthritis [73]. An effective treatment of the disease, ideally in specialized centers, is of paramount importance. It seems to be evident that surgical excision of bowel endometriosis should be considered the best cost-effective strategy in reducing disease-related costs linked to chronic pain, infertility, and long-term reduced quality of life; nonetheless, this evidence is still lacking [74]. Furthermore, intestinal surgery for endometriosis requires highly specialized interdisciplinary teams and resource allocation, which is linked to a high economic burden. Thus, limited economic and competency affordability, for example, in case of mini-invasive surgery or of nerve-sparing techniques, can limit accessibility to different techniques, particularly in countries with low economic resources.

## 4. Discussion

**Figure 2:**
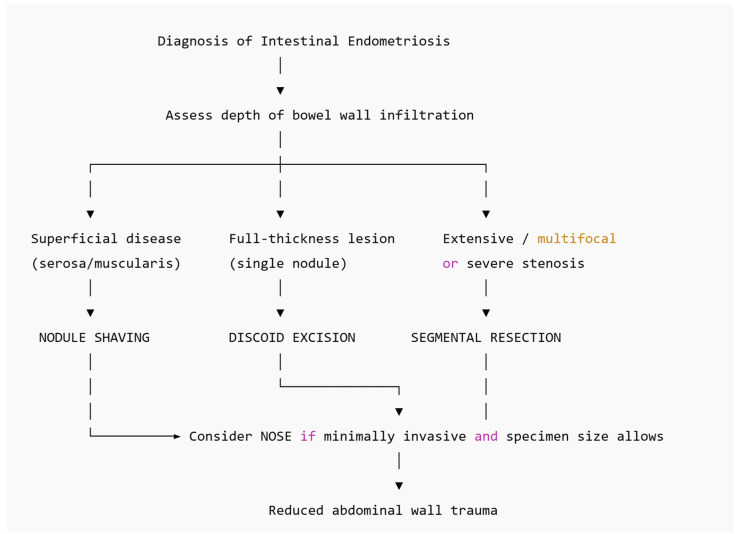
Decision-making flow diagram.

In this manuscript, we provided an extensive review of the current literature on DIE, specifically on bowel endometriosis. According to the current knowledge, the following three major procedures for the excision of bowel endometriosis have been proposed: nodule shaving, discoid excision, and segmental intestinal resection. The NOSE (natural orifice specimen extraction) technique can be applied for the removal of the specimen in case of discoid or segmental resection. Specific issues concerning the different techniques for bowel endometriosis surgery have been evaluated, including risks and advantages, gastrointestinal, bladder, and sexual function constraints, as well as quality-of-life-related issues. The most relevant indications, advantages, risks, and limitations of the different techniques are summarized in Table 1. A simplified decision-making flow diagram is proposed in Figure 2.

### 4.1. Bowel Endometriosis Management and Classification: Gaps to Be Addressed

As discussed above, for the variety of clinical presentations and severity of bowel endometriosis, a range of different surgical procedures have been proposed. Nevertheless, the evidence supporting the choice of a specific surgical intervention is limited by the absence of an accepted classification system to describe in detail the chosen surgical treatment. The lack of multicenter randomized controlled trials represents a relevant limitation and a call for future clinical research. Furthermore, a recognized list of relevant clinical and surgical outcomes is missing, thus hindering scientific and clinical progress. In particular, we want to stress the underrepresentation of fertility outcomes and the reporting on sexual function and quality of life. Altogether, without a unified framework, the ability to compare studies and determine the superiority of one surgical method over another remains a formidable challenge.

### 4.2. A Proposal for a New Classification

To fill these gaps, we propose a new classification system for bowel surgery. This will provide a more detailed description of the pathology and the procedure used to treat it, underpinning a better standardization of its management. It is based on the use of a letter that describes the dimension of the lesion, as well as of a number that describes the type of surgical technique, supplemented by a plus or minus sign to specify if vaginal opening was necessary for complete lesion resection (Table 2).

One of the most crucial criteria in guiding the choice of surgical technique is lesion size. It is well-recognized that lesion dimension has a major impact both on the clinical presentation of the disease, being implicated in the degree of bowel stenosis, as well as in the feasibility of some resection techniques, notably the discoid excision. In addition, the presence of multiple lesions, either multifocal or multicentric, will greatly influence these features and should be documented.

Importantly, the type of surgery chosen should be accurately documented. This will help to standardize the measure of the most relevant outcomes, including perioperative and long-term complications, as well as the intestinal functional outcomes and other parameters used for the evaluation of the quality of life of our patients. Concerning the nodule shaving technique, the degree of involvement of the shaving should be documented (confined to the serosa, involving the muscularis or even reaching the serosa). For discoid excision, it should be documented if the technique used was manual or mechanical or repeated excisions had to be performed in case of larger nodules. Concerning the segmental resection, the type of anastomosis (end-to-end vs. end-to-side anastomosis) should be noted. If a natural orifice specimen extraction (NOTE) technique was used, that should also be noted. Finally, as vaginal opening could affect the outcome, this should be documented.

### 4.3. Limitations and Future Work

Even though the clinical experience concerning the surgical treatment of bowel endometriosis is robust, there is limited randomized data that compare surgical techniques. This limits the possibility to draw firm conclusions on the different techniques, including advantages and drawbacks. However, our review underlines the heterogeneity of the outcomes evaluated and the lack of detailed data on the type of procedure used.

The introduction of this classification is expected to help researchers to perform a more focused follow-up concerning the different procedures that can be used in bowel endometriosis. It is not intended to replace the #Enzian classification, but rather to complete and implement it, as the same type of endometriosis lesion can be treated with different techniques. The proposed classification seems to be easy to use and susceptible to minor inter-observer variation. The evaluation and documentation of the number and dimension of the intestinal lesions is intraoperatively easy to perform by the surgeon once the nodule has been removed or shaved. The type of chosen surgery is easy to document as well. However, it is worth noting that the proposed classification system should be intended as a theory without current supporting data available. This classification should now be validated. Prospective cohort studies or inclusion into a multi-institutional registry is strongly advised.

## 5. Conclusions

Significant barriers in comparing surgical outcomes due to unclear definitions, lack of standardization, and incomplete reporting are some of the most relevant issues frequently encountered. To fill these gaps, we propose a new classification system for bowel surgery that describes the dimension and the number of the lesions, as well as the type of surgical technique used, supplemented by the information if vaginal opening was necessary for complete lesion resection. This proposition aims to open a discussion on this topic and boost focused research to evaluate the utility of a new classification in clinical practice.

## Figures and Tables

**Table 1:** Summary of the different techniques for bowel endometriosis surgery.

Procedure	Extent of Resection	Key Decision-Making Parameters	Advantages	Risks/Limitations	Typical Indications
Nodule Shaving	Superficial excision of serosa and muscularis; mucosa preserved	Nodule does not infiltrate mucosa; Limited bowel wall invasion; Desire to preserve bowel integrity; Minimal stenosis (<30–40%).	Lowest surgical morbidity; Preserves bowel function; Shorter operative time; Lower risk of leaks and strictures.	Higher risk of residual disease; Possible symptom recurrence Technically demanding to avoid mucosal injury.	Small, superficial nodules; Fertility-preserving approach; Patients with mild/moderate symptoms.
Discoid Excision	Full-thickness excision of bowel wall with circular closure or stapler	Single lesion; Full-thickness involvement; Limited diameter (<3 cm typically); Stenosis usually <50%.	Removes full-thickness disease; Preserves bowel length; Lower recurrence than shaving.	Risk of anastomotic leak (lower than segmental); Risk of stricture at excision site; Technical complexity.	Isolated deep nodule; Moderate bowel wall infiltration.
Segmental Intestinal Resection	Resection of affected bowel segment with end-to-end anastomosis	Multiple nodules; Long lesions (>3–4 cm); Severe stenosis (>50%); Multifocal or circumferential disease.	Most radical disease clearance; Lowest local recurrence rate.	Highest morbidity; Anastomotic leak risk; Bowel dysfunction (LARS, diarrhea, and constipation); Possible nerve injury.	Severe symptoms; Obstructive disease; Extensive or multifocal involvement.
NOSE (Specimen Extraction Technique)	No additional bowel resection; affects specimen retrieval only	Minimally invasive approach planned Specimen size compatible with transvaginal/transanal extraction; Patient anatomy suitable.	Avoids abdominal extraction incision; Less post-operative pain; Lower wound infection risk; Better cosmetic outcomes.	Not suitable for large specimens; Requires expertise; Theoretical contamination risk.	Adjunct to discoid or segmental resection; Selected laparoscopic cases.

**Table 2:** Proposal for a new standardized classification system.

Lesion Size
A: ≤3 cm
B: >3 cm
C: Multifocal lesions (>one bowel segment)
D: Multicentric lesions (>two lesions on the same bowel segment)
Surgical method
1 Nodule shaving
1.1 Serosal
1.2 Muscular
1.3 Submucosal
1.4 Intramucosal
2 Discoid excision
2.1 Manual
2.2 Mechanical
2.3 Repeated
3 Segmental resection
3.1 With end-to-end anastomosis
3.2 With end-to-side anastomosis
3.3 NOSE technique
Vaginal wall involvement
− Without vaginal opening
+ With vaginal opening

## Data Availability

No new data were created or analyzed in this study. Data sharing is not applicable to this article.

## Abbreviations

The following abbreviations are used in this manuscript:

DIE	Deep infiltrating endometriosis
NOTE	Natural orifice specimen extraction
rASRM	Revised American Society of Reproductive Medicine
miRNA	Micro ribonucleic acid
DNA	Deoxyribonucleic acid
LARS	Low anterior rectal resection syndrome

## References

[1] Parasar P., Ozcan P., Terry K.L. (2017). Endometriosis: Epidemiology, Diagnosis and Clinical Management. Curr. Obstet. Gynecol. Rep..

[2] Giudice L.C., Kao L.C. (2004). Endometriosis. Lancet Lond. Engl..

[3] Taylor H.S., Kotlyar A.M., Flores V.A. (2021). Endometriosis is a chronic systemic disease: Clinical challenges and novel innovations. Lancet.

[4] Horne A.W., Missmer S.A. (2022). Pathophysiology, diagnosis, and management of endometriosis. BMJ.

[5] Lamceva J., Uljanovs R., Strumfa I. (2023). The Main Theories on the Pathogenesis of Endometriosis. Int. J. Mol. Sci..

[6] Zondervan K.T., Becker C.M., Koga K., Missmer S.A., Taylor R.N., Viganò P. (2018). Endometriosis. Nat. Rev. Dis. Primer.

[7] Page M.J., McKenzie J.E., Bossuyt P.M., Boutron I., Hoffmann T.C., Mulrow C.D., Shamseer L., Tetzlaff J.M., Akl E.A., Brennan S.E. (2021). The PRISMA 2020 statement: An updated guideline for reporting systematic reviews. BMJ.

[8] Bokor A., Hudelist G., Dobó N., Dauser B., Farella M., Brubel R., Tuech J.-J., Roman H. (2021). Low anterior resection syndrome following different surgical approaches for low rectal endometriosis: A retrospective multicenter study. Acta Obstet. Gynecol. Scand..

[9] Maguire B., DeMaio A., O’Neill A., Clancy C. (2025). A quality-of-life meta-analysis comparing pre- and postoperative symptoms in women undergoing colorectal resection for deep infiltrating endometriosis. Color. Dis. Off. J. Assoc. Coloproctol. G. B. Irel..

[10] Ceccaroni M., Ceccarello M., Raimondo I., Roviglione G., Clarizia R., Bruni F., Mautone D., Manzone M., Facci E., Rettore L. (2023). “A Space Odyssey” on Laparoscopic Segmental Rectosigmoid Resection for Deep Endometriosis: A Seventeen-year Retrospective Analysis of Outcomes and Postoperative Complications among 3050 Patients Treated in a Referral Center. J. Minim. Invasive Gynecol..

[11] Ferrero S., Stabilini C., Barra F., Clarizia R., Roviglione G., Ceccaroni M. (2021). Bowel resection for intestinal endometriosis. Best Pract. Res. Clin. Obstet. Gynaecol..

[12] Meuleman C., Tomassetti C., Wolthuis A., Van Cleynenbreugel B., Laenen A., D’Hoore A., D’Hooghe T. (2015). Increasing the Quality of Surgery for Deep Endometriosis Should Be Based on Homogenous Clinical Patient Phenotype, Surgical Experience, and Standardized Outcome Reporting in Multicenter Multisurgeon Prospective Trials. Ann. Surg..

[13] American College of Obstetricians and Gynecologists (2010). Practice bulletin no. 114: Management of endometriosis. Obstet. Gynecol..

[14] Somigliana E., Infantino M., Candiani M., Vignali M., Chiodini A., Busacca M., Vignali M. (2004). Association rate between deep peritoneal endometriosis and other forms of the disease: Pathogenetic implications. Hum. Reprod. Oxf. Engl..

[15] Remorgida V., Ferrero S., Fulcheri E., Ragni N., Martin D.C. (2007). Bowel endometriosis: Presentation, diagnosis, and treatment. Obstet. Gynecol. Surv..

[16] Campagnacci R., Perretta S., Guerrieri M., Paganini A.M., De Sanctis A., Ciavattini A., Lezoche E. (2005). Laparoscopic colorectal resection for endometriosis. Surg. Endosc..

[17] Shafrir A.L., Farland L.V., Shah D.K., Harris H.R., Kvaskoff M., Zondervan K., Missmer S.A. (2018). Risk for and consequences of endometriosis: A critical epidemiologic review. Best Pract. Res. Clin. Obstet. Gynaecol..

[18] Zondervan K.T., Becker C.M., Missmer S.A. (2020). Endometriosis. N. Engl. J. Med..

[19] Gordts S., Koninckx P., Brosens I. (2017). Pathogenesis of deep endometriosis. Fertil. Steril..

[20] Sapkota Y., Steinthorsdottir V., Morris A.P., Fassbender A., Rahmioglu N., De Vivo I., Buring J.E., Zhang F., Edwards T.L., Jones S. (2017). Meta-analysis identifies five novel loci associated with endometriosis highlighting key genes involved in hormone metabolism. Nat. Commun..

[21] Cardoso J.V., Perini J.A., Machado D.E., Pinto R., Medeiros R. (2020). Systematic review of genome-wide association studies on susceptibility to endometriosis. Eur. J. Obstet. Gynecol. Reprod. Biol..

[22] Rahmioglu N., Mortlock S., Ghiasi M., Møller P.L., Stefansdottir L., Galarneau G., Turman C., Danning R., Law M.H., Sapkota Y. (2023). The genetic basis of endometriosis and comorbidity with other pain and inflammatory conditions. Nat. Genet..

[23] Ducreux B., Patrat C., Firmin J., Ferreux L., Chapron C., Marcellin L., Parpex G., Bourdon M., Vaiman D., Santulli P. (2025). Systematic review on the DNA methylation role in endometriosis: Current evidence and perspectives. Clin. Epigenet..

[24] Mortlock S., Houshdaran S., Kosti I., Rahmioglu N., Nezhat C., Vitonis A.F., Andrews S.V., Grosjean P., Paranjpe M., Horne A.W. (2023). Global endometrial DNA methylation analysis reveals insights into mQTL regulation and associated endometriosis disease risk and endometrial function. Commun. Biol..

[25] Antonio L.G.L., Meola J., Rosa-E-Silva A.C.J.d.S., Nogueira A.A., Candido Dos Reis F.J., Poli-Neto O.B., Rosa-E-Silva J.C. (2023). Altered Differential Expression of Genes and microRNAs Related to Adhesion and Apoptosis Pathways in Patients with Different Phenotypes of Endometriosis. Int. J. Mol. Sci..

[26] Keckstein J., Saridogan E., Ulrich U.A., Sillem M., Oppelt P., Schweppe K.W., Krentel H., Janschek E., Exacoustos C., Malzoni M. (2021). The #Enzian classification: A comprehensive non-invasive and surgical description system for endometriosis. Acta Obstet. Gynecol. Scand..

[27] Vercellini P., Frontino G., Pietropaolo G., Gattei U., Daguati R., Crosignani P.G. (2004). Deep endometriosis: Definition, pathogenesis, and clinical management. J. Am. Assoc. Gynecol. Laparosc..

[28] Donnez O., Donnez J. (2021). Deep endometriosis: The place of laparoscopic shaving. Best Pract. Res. Clin. Obstet. Gynaecol..

[29] Xu S., Zhang Y., Ye P., Huang Q., Wang Y., Zhang Y., Yang C., Ding J. (2025). Global, regional, and national burden of endometriosis among women of childbearing age from 1990 to 2021: A cross-sectional analysis from the 2021 global burden of disease study. Int. J. Surg. Lond. Engl..

[30] Tuominen A., Saavalainen L., Niinimäki M., Gissler M., But A., Härkki P., Heikinheimo O. (2023). First live birth before surgical verification of endometriosis-a nationwide register study of 18,324 women. Hum. Reprod. Oxf. Engl..

[31] Mappa I., Page Z.P., Di Mascio D., Patelli C., D’Antonio F., Giancotti A., Gebbia F., Mariani G., Cozzolino M., Muzii L. (2024). The Effect of Endometriosis on In Vitro Fertilization Outcomes: A Systematic Review and Meta-Analysis. Healthcare.

[32] Pashkunova D., Darici E., Senft B., Bokor A., Hudelist T., Tammaa A., Hudelist G. (2024). Lesion size and location in deep infiltrating bowel endometriosis: Correlation with gastrointestinal dysfunction and pain. Acta Obstet. Gynecol. Scand..

[33] As-Sanie S., Mackenzie S.C., Morrison L., Schrepf A., Zondervan K.T., Horne A.W., Missmer S.A. (2025). Endometriosis: A Review. JAMA.

[34] De Cicco C., Corona R., Schonman R., Mailova K., Ussia A., Koninckx P. (2011). Bowel resection for deep endometriosis: A systematic review. BJOG Int. J. Obstet. Gynaecol..

[35] Darici E., Bokor A., Miklos D., Pashkunova D., Rath A., Hudelist G. (2025). Gastrointestinal function and pain outcomes following segmental resection or discoid resection for low rectal endometriosis. Wien. Klin. Wochenschr..

[36] Namazov A., Kathurusinghe S., Mehdi E., Merlot B., Prosszer M., Tuech J.J., Marpeau L., Roman H. (2022). Evolution of Bowel Complaints after Laparoscopic Endometriosis Surgery: A 1497 Women Comparative Study. J. Minim. Invasive Gynecol..

[37] Iversen M.L., Seyer-Hansen M., Forman A. (2017). Does surgery for deep infiltrating bowel endometriosis improve fertility? A systematic review. Acta Obstet. Gynecol. Scand..

[38] Vallée A., Ceccaldi P.-F., Carbonnel M., Horsman S., Murtada R., Moawad G., Feki A., Ayoubi J.-M. (2025). Comparative pregnancy rate after colorectal resection versus other surgical procedures for deep infiltrating rectal endometriosis: A systematic review and meta-analysis. Sci. Rep..

[39] Akladios C., Faller E., Afors K., Puga M., Albornoz J., Redondo C., Leroy J., Wattiez A. (2014). Totally laparoscopic intracorporeal anastomosis with natural orifice specimen extraction (NOSE) techniques, particularly suitable for bowel endometriosis. J. Minim. Invasive Gynecol..

[40] Elftmann T.D., Nelson H., Ota D.M., Pemberton J.H., Beart R.W. (1994). Laparoscopic-assisted segmental colectomy: Surgical techniques. Mayo Clin. Proc..

[41] Crestani A., Merlot B., Goualard P.-H., Grigoriadis G., Chanavaz Lacheray I., Dennis T., Roman H. (2024). Bowel endometriosis: Surgical customization is demanding. Best Pract. Res. Clin. Obstet. Gynaecol..

[42] Landi S., Ceccaroni M., Perutelli A., Allodi C., Barbieri F., Fiaccavento A., Ruffo G., McVeigh E., Zanolla L., Minelli L. (2006). Laparoscopic nerve-sparing complete excision of deep endometriosis: Is it feasible?. Hum. Reprod..

[43] O’Brien L., Morarasu S., Morarasu B.C., Neary P.C., Musina A.M., Velenciuc N., Roata C.E., Dimofte M.G., Lunca S., Raimondo D. (2023). Conservative surgery versus colorectal resection for endometriosis with rectal involvement: A systematic review and meta-analysis of surgical and long-term outcomes. Int. J. Color. Dis..

[44] Tsuei A., Nezhat F., Amirlatifi N., Najmi Z., Nezhat A., Nezhat C. (2025). Comprehensive Management of Bowel Endometriosis: Surgical Techniques, Outcomes, and Best Practices. J. Clin. Med..

[45] Ceccaroni M., Clarizia R., Mussi E.A., Stepniewska A.K., De Mitri P., Ceccarello M., Ruffo G., Bruni F., Rettore L., Surico D. (2022). “The Sword in the Stone”: Radical excision of deep infiltrating endometriosis with bowel shaving-a single-centre experience on 703 consecutive patients. Surg. Endosc..

[46] Bendifallah S., Vesale E., Daraï E., Thomassin-Naggara I., Bazot M., Tuech J.-J., Abo C., Roman H. (2020). Recurrence after Surgery for Colorectal Endometriosis: A Systematic Review and Meta-analysis. J. Minim. Invasive Gynecol..

[47] Roman H., Moatassim-Drissa S., Marty N., Milles M., Vallée A., Desnyder E., Stochino Loi E., Abo C. (2016). Rectal shaving for deep endometriosis infiltrating the rectum: A 5-year continuous retrospective series. Fertil. Steril..

[48] Donnez O., Roman H. (2017). Choosing the right surgical technique for deep endometriosis: Shaving, disc excision, or bowel resection?. Fertil. Steril..

[49] Klapczynski C., Derbal S., Braund S., Coget J., Forestier D., Seyer-Hansen M., Tuech J.-J., Roman H. (2021). Evaluation of functional outcomes after disc excision of deep endometriosis involving low and mid rectum using standardized questionnaires: A series of 80 patients. Color. Dis. Off. J. Assoc. Coloproctol. G. B. Irel..

[50] Namazov A., Kathurusinghe S., Marabha J., Merlot B., Forestier D., Hennetier C., Tuech J.-J., Roman H. (2020). Double Disk Excision of Large Deep Endometriosis Nodules Infiltrating the Low and Mid Rectum: A Pilot Study of 20 Cases. J. Minim. Invasive Gynecol..

[51] Farella M., Tuech J.-J., Bridoux V., Coget J., Chati R., Resch B., Marpeau L., Roman H. (2021). Surgical Management by Disk Excision or Rectal Resection of Low Rectal Endometriosis and Risk of Low Anterior Resection Syndrome: A Retrospective Comparative Study. J. Minim. Invasive Gynecol..

[52] Malzoni M., Di Giovanni A., Exacoustos C., Lannino G., Capece R., Perone C., Rasile M., Iuzzolino D. (2016). Feasibility and Safety of Laparoscopic-Assisted Bowel Segmental Resection for Deep Infiltrating Endometriosis: A Retrospective Cohort Study With Description of Technique. J. Minim. Invasive Gynecol..

[53] Millochau J.-C., Stochino-Loi E., Darwish B., Abo C., Coget J., Chati R., Tuech J.-J., Roman H. (2018). Multiple Nodule Removal by Disc Excision and Segmental Resection in Multifocal Colorectal Endometriosis. J. Minim. Invasive Gynecol..

[54] Kar E., Philip C.E., Eskandar K., Polat I., Bastu E. (2024). Natural Orifice Specimen Extraction as a Promising Alternative for Minilaparotomy in Bowel Resection Due to Endometriosis: A Systematic Review and Meta-Analysis. J. Minim. Invasive Gynecol..

[55] Malzoni M., Di Giovanni A., Coppola M., Iuzzolino D., Casarella L., Rasile M., Falcone F. (2025). Total Laparoscopic Segmental Resection With Transanal Natural Orifice Specimen Extraction for Treatment of Colorectal Endometriosis: Descriptive Analysis From the TrEnd Study Database. J. Minim. Invasive Gynecol..

[56] Dobó N., Márki G., Hudelist G., Csibi N., Brubel R., Ács N., Bokor A. (2023). Laparoscopic natural orifice specimen extraction colectomy versus conventional laparoscopic colorectal resection in patients with rectal endometriosis: A randomized, controlled trial. Int. J. Surg. Lond. Engl..

[57] Abo C., Moatassim S., Marty N., Saint Ghislain M., Huet E., Bridoux V., Tuech J.J., Roman H. (2018). Postoperative complications after bowel endometriosis surgery by shaving, disc excision, or segmental resection: A three-arm comparative analysis of 364 consecutive cases. Fertil. Steril..

[58] Emmertsen K.J., Laurberg S. (2012). Low anterior resection syndrome score: Development and validation of a symptom-based scoring system for bowel dysfunction after low anterior resection for rectal cancer. Ann. Surg..

[59] Fuchs K.-H., Musial F., Retzbach L., Hann A., Meining A. (2023). Quality of life in benign colorectal disease-a review of the assessment with the Gastrointestinal Quality of Life Index (GIQLI). Int. J. Color. Dis..

[60] Vesale E., Roman H., Moawad G., Benoit L., Touboul C., Darai E., Bendifallah S. (2020). Voiding Dysfunction after Colorectal Surgery for Endometriosis: A Systematic Review and Meta-analysis. J. Minim. Invasive Gynecol..

[61] Madar A., Crestani A., Eraud P., Spiers A., Constantin A., Chiche F., Furet E., Collinet P., Touboul C., Merlot B. (2025). Voiding dysfunction after surgery for colorectal deep infiltrating endometriosis: An updated systematic review and meta-analysis. Updat. Surg..

[62] Garratt A.M., Torgerson D.J., Wyness J., Hall M.H., Reid D.M. (1995). Measuring sexual functioning in premenopausal women. Br. J. Obstet. Gynaecol..

[63] Wiegel M., Meston C., Rosen R. (2005). The female sexual function index (FSFI): Cross-validation and development of clinical cutoff scores. J. Sex Marital Ther..

[64] Pluchino N., Wenger J.-M., Petignat P., Tal R., Bolmont M., Taylor H.S., Bianchi-Demicheli F. (2016). Sexual function in endometriosis patients and their partners: Effect of the disease and consequences of treatment. Hum. Reprod. Update.

[65] Capezzuoli T., Maseroli E., Barra F., Vannuccini S., Vignozzi L., De Mitri P., Baggio S., Ceccaroni M., Petraglia F. (2023). Endometriosis and sexual disorders: The effect of surgical and medical treatment, a multicentre cross-sectional study. F1000Research.

[66] Maulenkul T., Kuandyk A., Makhadiyeva D., Dautova A., Terzic M., Oshibayeva A., Moldaliyev I., Ayazbekov A., Maimakov T., Saruarov Y. (2024). Understanding the impact of endometriosis on women’s life: An integrative review of systematic reviews. BMC Womens Health.

[67] Riemma G., De Franciscis P., La Verde M., Ravo M., Fumiento P., Fasulo D.D., Della Corte L., Ronsini C., Torella M., Cobellis L. (2023). Impact of the hemostatic approach after laparoscopic endometrioma excision on ovarian reserve: Systematic review and network meta-analysis of randomized controlled trials. Int. J. Gynaecol. Obstet. Off. Organ Int. Fed. Gynaecol. Obstet..

[68] Ata B., Turkgeldi E., Seyhan A., Urman B. (2015). Effect of hemostatic method on ovarian reserve following laparoscopic endometrioma excision; comparison of suture, hemostatic sealant, and bipolar dessication. A systematic review and meta-analysis. J. Minim. Invasive Gynecol..

[69] Hsu J.-Z., Ding D.-C. (2025). Association between endometriosis and pregnancy complications: A nationwide retrospective analysis (2000–2021). Eur. J. Obstet. Gynecol. Reprod. Biol..

[70] Šalamun V., Riemma G., Sirc T., Vrtacnik Bokal E., Ban Frangež H. (2024). Pregnancy and Neonatal Outcomes in Women Treated for Bowel Endometriosis: A Seven-Year Single-Centre Retrospective Matched Cohort Study. J. Clin. Med..

[71] Busnelli A., Di Simone N., Somigliana E., Greppi D., Cirillo F., Bulfoni A., Inversetti A., Levi-Setti P.E. (2024). Untangling the independent effect of endometriosis, adenomyosis, and ART-related factors on maternal, placental, fetal, and neonatal adverse outcomes: Results from a systematic review and meta-analysis. Hum. Reprod. Update.

[72] Latif S., Khanjani S., Saridogan E. (2024). Endometriosis and In Vitro Fertilization. Med. Kaunas Lith..

[73] Simoens S., Dunselman G., Dirksen C., Hummelshoj L., Bokor A., Brandes I., Brodszky V., Canis M., Colombo G.L., DeLeire T. (2012). The burden of endometriosis: Costs and quality of life of women with endometriosis and treated in referral centres. Hum. Reprod. Oxf. Engl..

[74] de Koning R., Cantineau A.E.P., van der Tuuk K., De Bie B., Groen H., van den Akker-van Marle M.E., Nap A.W., Maas J.W.M., Jansen F.W., Twijnstra A.R.H. (2024). The (cost-) effectiveness of Surgical excision of Colorectal endometriosis compared to ART treatment trajectory (TOSCA study)—A study protocol. Reprod. Fertil..

